# Potential Effects of Nutrient Profiles on Nutrient Intakes in the Netherlands, Greece, Spain, USA, Israel, China and South-Africa

**DOI:** 10.1371/journal.pone.0014721

**Published:** 2011-02-23

**Authors:** Annet J. C. Roodenburg, Anke Schlatmann, Mariska Dötsch-Klerk, Robert Daamen, Jie Dong, Marta Guarro, Margarita Stergiou, Nazeeia Sayed, Eunice Ronoh, Léon Jansen, Jacob C. Seidell

**Affiliations:** 1 Department of Health Sciences, VU University Amsterdam, Amsterdam, The Netherlands; 2 Unilever Research and Development, Vlaardingen, The Netherlands; 3 Unilever Benelux, Rotterdam, The Netherlands; 4 Unilever Discover, Shanghai, China; 5 Unilever Spain, Barcelona, Spain; 6 Unilever Greece, Athens, Greece; 7 Unilever South Africa, Durban, South Africa; 8 Unilever Israel, Tel Aviv, Israel; 9 Choices International Foundation, Brussels, Belgium; AgroParisTech, France

## Abstract

**Introduction:**

Nutrient profiling is defined as the science of categorising foods based on their nutrient composition. The Choices Programme is a nutrient profile system with criteria that determine whether foods are eligible to carry a “healthier option” stamp. The Daily Menu Method which has been developed to evaluate these criteria is described here. This method simulates the change in calculated nutrient intakes which would be the result of consumers changing their diets in favour of food products that comply with the criteria.

**Methods:**

Average intakes of energy, trans fatty acids (TFA), saturated fatty acids (SAFA), sodium, added sugar and fibre were derived from dietary intake studies and food consumption surveys of 7 countries: The Netherlands, Greece, Spain, the USA, Israel, China and South Africa. For each of the key nutrients, these average intakes were translated into three Typical Daily Menus per country. Average intakes based on these three menus were compared with average intakes from three Choices Daily Menus. To compose the Choices Menus, foods from the Typical Menus that did not comply with the Choices criteria were replaced with foods that did comply and are available on the market.

**Results:**

Comparison of intakes from the Choices Menus with the survey data showed that calculated intakes of energy, SAFA, TFA, sodium and added sugar were reduced. Fibre intakes were increased. The size of the effect differed per country.

**Conclusion:**

The Daily Menu Method is a useful means to predict the potential effects of nutrient profiles such as the Choices criteria, on daily nutrient intakes. The method can be applied internationally and confirms that the criteria of the Choices Programme are in line with the aim of the programme: to improve nutrient intakes in the direction of the recommendations.

## Introduction

Globally, dietary intakes of trans fatty acids (TFA), saturated fatty acids (SAFA), sodium and sugar exceed the recommendations [1]. Therefore the World Health Organisation's (WHO) Global Strategy on Diet, Physical Activity and Health recommended the private sector to limit the levels of TFA, SAFA, salt and free sugars in existing products [2].

A way to achieve this goal is by the development of nutrient profiles with criteria that can be used as targets for food reformulation. Various nutrient profiling systems exist and they have been developed for different purposes such as claims eligibility, advertising, signposting and food reformulation [3]–[10]. However, there is no gold standard. In a recent publication [11] a conceptual framework was offered for validating nutrient profiles. In addition Drewnowski and Fulgoni signalled that validation of nutrient profiling systems is of highest research importance [12]. Thus far, methods to evaluate nutrient profiles are aimed at determining whether the foods are categorized correctly as “healthier” or “less healthy”. An alternative way of approaching evaluation of nutrient profiles is to estimate whether they are suitable for their purpose, for example their potential to influence daily nutrient intakes. The Daily Menu Method described in this paper has been developed to test the criteria for the Choices Programme, by predicting the effect on nutrient intakes [13], [14].

The Choices Programme is an international applicable nutrient profiling system with criteria that determine whether foods are eligible to carry a “healthier option” stamp. The aims of the Choices Programme are to stimulate product reformulation and to help consumers by making healthier choices easier to identify. For the development of the nutrient profiles for Choices, the generic criteria for energy and the key nutrients (TFA, SAFA, sodium, added sugar and fiber) were derived from international nutrient intake recommendations for daily diets [1], [9]. The ultimate goal is to meet these recommendations for population intakes. To evaluate the Choices Programme, it is hypothesized that if consumers choose food products that comply with the Choices criteria, the calculated daily intake of the key nutrients should improve in the direction of the nutrient intake recommendations. To determine this, an evaluation method based on national dietary surveys and daily diets (the Daily Menu Method [14]) has been described in more detail in the present paper and has been applied to various countries across the world.

## Methods

### The Daily Menu Method

**Figure 1:**
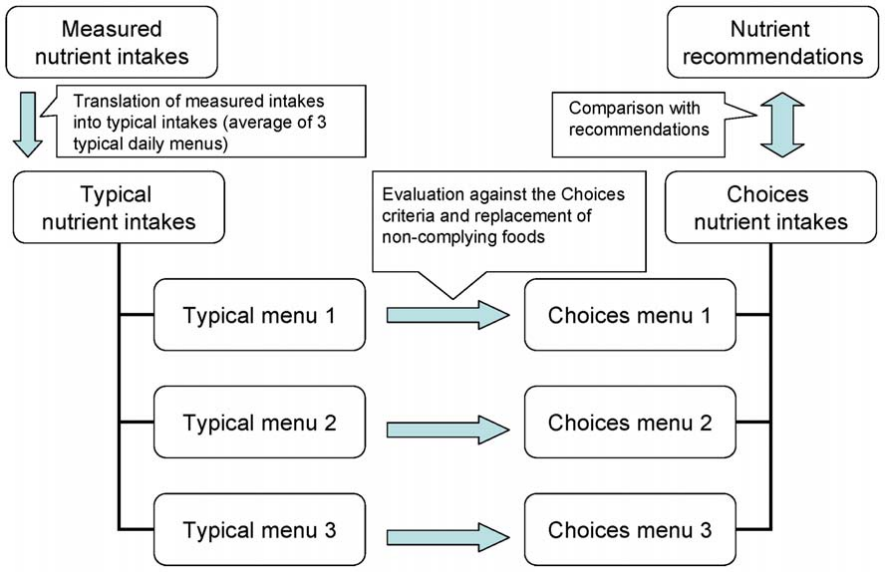
Description of Daily Menu Method. Average intakes of energy SAFA, TFA, sodium, sugar and fibre were derived from dietary intake studies and food consumption surveys. These average intakes were translated into three Typical Daily Menus per country. Average intakes based on these three menus were compared with average intakes from three Choices Daily Menus. To compose the Choices Menus, foods from the Typical Menus that were not complying with the Choices criteria were replaced with foods that did comply and are available on the market.

The Daily Menu Method is visualized in Figure 1. Nutrient intakes based on Typical Daily Menus are compared with intakes from Choices Daily Menus in which regular foods that do not comply with the Choices criteria have been replaced by foods that do comply. The application of this method for the Netherlands [14], [15] is summarised in Table 1. The Daily Menu Method was applied to various other countries: Spain, Greece, USA, China, Israel and South Africa. This work was carried out from January 2007 until May 2009.

**Table 1:** Daily Menu Method: Example from The Netherlands [14], [15].

Nutrient	International dietary recommendations*	Daily nutrient intakes based on National survey†	Daily nutrient intakes Typical Menus‡	Daily nutrient intakes Choices Menus§
**Energy**	2000 kcal/d	2190 kcal	2122 kcal	1788 kcal
**SAFA**	< 10 en%	14.2 en%	15.4 en%	8.4 en%
**TFA**	< 1 en%	1.7 en%	1.2 en%	0.1 en%
**Sodium**	< 2400 mg/d	2785 mg	2753 mg	2347 mg
**Sugar**	< 10 en% free sugar	15.5 en% added sugar	13.0 en% added sugar	5.8 en% added sugar
**Fibre**	> 25 g/d	21 g	18 g	25 g

Free sugar  =  added sugar; SAFA: saturated fatty acids; TFA: trans fatty acid.

*Recommendations for SAFA, TFA and free sugars are derived from WHO/FAO [1] and recommendation for sodium is derived from various other references [16]–[20].

† Derived from Dutch National Dietary Survey 1998 [15]; A translation for total sugars to added sugars has been applied by assuming that in general two-thirds of total sugars are delivered by added sugars.

‡ Typical Daily Menu  =  average of three Typical Menus based on the Dutch National Dietary Survey 1998 [15].

§ Choices Menu  =  same menu as ‘Typical Menu’ but with replacing regular products (not meeting Choices qualifying criteria) by Choices compliant products.

### Step 1. International nutrient recommendations

International nutrient recommendations as shown in the first column of Table 1, formed the basis of the development of the Choices criteria defined in 2007 [1], [9]. Whereas most international nutrient recommendations were derived from the Joint WHO/FAO Consultation on Diet, Nutrition, and the Prevention of Chronic Diseases [1], for sodium, a slightly less stringent recommendation of 2400 mg/d was chosen which is used by e.g Eurodiet [16], the UK [17], Germany, Austria and Switzerland [18], The Netherlands [19] and Portugal [20].

### Step 2. Actual intakes of the key nutrients based on dietary surveys

Daily Menu Method was applied in an international context. The actual key nutrient intakes for the Netherlands are shown in the second column of Table 1. The Dietary intake data were derived from a selection of 7 countries on 4 continents. Ideally these data should be representative for the whole population. However, the availability of the data for the actual key nutrient intakes in the specific countries determined its completeness. This is shown in Table 2, which gives details of the dietary intake data that were used. Some countries, such as The Netherlands, The United States of America and Israel [15], [21]–[23] have national programmes with comprehensive, cross-sectional information on the nutrient intake of the population. In order to arrive at actual key nutrient intake data from countries such as Spain [26]–[28], Greece [28]–[30], China [24], [25] and South Africa [31], [32], it was necessary to use several studies, although this still resulted in incomplete data (data were only available for a selection of the nutrients or for a selection of the population). In some cases, surveys only have information on foods and lack information on nutrient intakes [27], [31]. These surveys were used as information sources on dietary habits for the compilation of the Typical Menus. Added sugar intake was missing for most countries, except for the US [22]. For the Netherlands, added sugar intake was estimated based on the assumption (for the UK) that 2/3 of total sugar intake is approximately the same as added sugar [5],[33]. Reported intakes of sodium for The Netherlands [15], Greece [30] and Israel [23] did not include sodium from discretionary (table) salt. Table salt was measured in the survey of the US [21]. In South Africa [34] and Spain [26] sodium intake was measured by 24 h urinary excretion, which gives a more complete estimate of daily sodium intake. For China, data from the National Nutrition and Health Survey in 2002 were used [24]. Dietary intake data were collected for 68 962 subjects aged 2 to 70+. We have only used information on the urban population (21103 subjects). Discretionary salt intake in China was reported separately: 10.9 g/day for the urban population, which is equivalent to 4235 mg sodium (Table 2).

**Table 2:** Overview of nutrient intake data sources for the various countries.

Country	Survey	Year of data collection	Nutrients	Study population	n	Dietary assessment method
**Netherlands**	National Survey [15], [19]	1998	All	All ages	5958	2 day dietary record
**Greece**	Greek Epic study [29]	1994–1999	Energy & SAFA	Adults aged 20–86	20822	Food frequency questionnaire (FFQ)
	Transfair study [28]	1995	TFA	Adults aged 23–64	248	1 day 24 h recall
	Survey university of Crete [30]	1989–2001	Sodium & fibre	Medical students aged 20–24	951	1 day 24 h recall
**Spain**	Intersalt study [26]	1986	Sodium	Adults aged 20–59	400	24 h urinary sodium excretion
	Spanish Food panel [27]	2006	Energy & fibre	HouseholdsCatering establishmentsInstitutions	6000700200	Bar code scanner
	Transfair study [28]	1991	SAFA & TFA	All aged 1–74	3000	7 day dietary record on household level
**US**	National Health and Nutrition Examination Survey (NHANES) [21]	1999–2000	Energy, sodium & SAFA	All ages	8604	1 day 24 h recall
	NHANES III [22]	1988–1994	Added sugar	All ages	25820	1 day 24 h recall
	Continuing Survey of Food Intakes by Individuals (CSFII) [22]	(1994–1996, 1998)	Fibre	All ages	21035	1 day 24 h recall
**China**	National Survey [24]	2002	Energy, sodium & fibre	Urban population, all ages	21103	3 day 24 h recall; food weighted record on household level
	INTERMAP study [25]	1997–1999	SAFA & TFA	Adults aged 40–59	839	4 day 24 h recall
**Israel**	National Survey [23]	1999–2001	Energy, SAFA, sodium & fibre	Jews and Arabs (urban and rural) aged 25–60	3246	1 day 24 h recall
**South Africa**	Secondary analysis of various surveys [31]	1983–2000	Energy	All aged 10+	> 5000	1 day 24 h recall, FFQ
	Study on diet and blood pressure [34]	2002	Sodium	Adults aged 20–65 black urban	110	24 h urinary sodium excretion
	THUSA study [32]	1996–1998	SAFA, TFA & fibre	Women aged 15–80	1008	FFQ

SAFA: saturated fatty acid; TFA: trans fatty acid; n: number of subjects; FFQ: food frequency questionnaire.

### Step 3. Translation of measured nutrient intake data into three Typical Daily Menus

Nutrition surveys produce nutrient intake data that are representative for the study population. Usually information on foods most commonly consumed is included as well. All this information was used to compose three Typical Daily Menus, representative of the dietary intake of a healthy adult. It was decided to compose three menus, to optimally simulate average daily nutrient intakes [35]. This was done manually by a nutritionist or dietician who knew the dietary habits of the country's population.

Composing the three Typical Daily Menus was an iterative and time consuming process. The average nutrient intakes from the three Typical Daily Menus need to approximate to the nutrient intakes from the national survey. Therefore it was necessary to adapt the menus during this process of translation with certain foods being replaced by others in order to better simulate the intakes from the survey. The maximum allowed deviation from the actual intakes was set at 20%. The nutritionist or dietician was instructed to adhere as closely as possible to the dietary habits of the country. The resulting menus are available as Supporting Information (Tables S1, S2, S3, S4, S5, S6, S7).

Ideally the food composition data were derived from the national food composition databases for each of the countries [36]–[42]. However when these data were not available for one or more of the key nutrients other data sources were used, such as food composition databases from other countries [36], [43]–[45]. These decisions were documented and are available as Supporting Information (Table S8).

In countries such as China and South Africa, where diets differ depending on where people live (in rural or urban areas) urban daily menus were composed. These menus contain less home-made dishes and more processed foods. It was anticipated that intakes of urban populations would be more affected by substitution with foods complying with the Choices criteria. In South Africa diets differ also between population groups. Here the focus was on the eating patterns of black urban women, because of data availability (women) and because most of South Africa's population is black and lives in urban areas. In China and South Africa, the intakes of discretionary salt were not incorporated into the Typical Daily Menus.

### Step 4. The Choices Daily Menus

After evaluation of the menus against the Choices criteria, non-compliant food products were replaced by those that did comply and were available on the market in the respective country. If it was not possible to find a suitable replacement food product in the food composition table, various producers' websites and the Global New Product Database [46] were used. In several cases it was not possible to find a replacement food product in that country (e.g egg, white rice, cereals, specific meat and cheese products). One example of a single Dutch Daily menu is given in Table 3. Detailed information on all the menus is available as Supporting Information (Tables S1, S2, S3, S4, S5, S6, S7).

**Table 3:** Example of one single Daily Menu from The Netherlands.*

Typical Menu		Choices Menu	
	Portion (g)		Portion (g)
**Breakfast**		**Breakfast**	
2× wholemeal bread	70	2× wholemeal bread	70
*1× chocolate sprinkels*	15	1× jam without sugar	15
*1× 48+ cheese*	20	1×30+ reduced fat cheese	20
*2×60% margarine fat <17g SAFA*	10	2× low fat margarine	10
1× semi skimmed milk	150	1× semi skimmed milk	150
1× tea	150	1× tea	150
**In between**		**In between**	
1× banana	100	1× banana	100
*1× cookies average*	10	1 gingerbread less sugar	23
2× coffee	300	2× coffee	300
*2× evaporated milk (for coffee)*	16	2× evaporated milk	16
**Lunch**		**Lunch**	
*1× white bread (bun)*	50		
*1× kroket (ragou fried in breadcrumbs)*	70	toasty sandwich turkey-spinach	105
*1× mustard*	5		
*1× wholemeal bread*	35	1× wholemeal bread	35
*1× 48+cheese*	20	1× 30+ reduced fat cheese	20
*2×60% margarine fat <17g SAFA*	10	1× low fat margarine	5
*1× drink, Milk and fruit (milk product)*	150	1× drink, milk and fruit, light	150
**In between**		**In between**	
*1× slice of cake*	25	1× biscuit	25
1× coffee	150	1× coffee	150
*1× evaporated milk (for coffee)*	8	1× evaporated milk	8
*1× soft drink*	150	1× ice tea (50% less sugar)	150
**Diner**		**Diner**	
*meatball (beef) prepared*	75	1 chicken filet unprocessed	75
cauliflower	130	cauliflower	130
potatoes	100	potatoes	130
*margarine*	15	cooking fat	15
*1× serving custard*	150	yoghurt	150
**In between**		**In between**	
1× beer	200	1× beer	200
*2× soft drink*	300	2× drink, light apple/peach	300
1× tea	150	1× tea	150
*1× piece of chocolate (average)*	5	1× biscuit	25
**Legend Font:**Normal: Food complying to Choices criteria;*Italics*: *Food not complying to Choices criteria*			

SAFA: saturated fatty acid.

*For every country three Daily Menus were prepared. Details on all the menus are available as Supporting Information (Tables S1, S2, S3, S4, S5, S6, S7).

**Figure 2:**
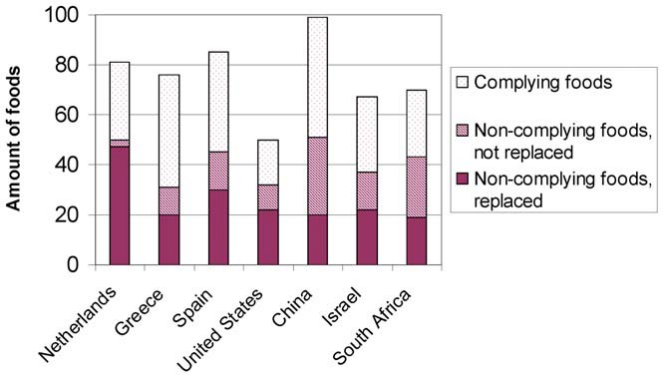
The amount of foods in the Typical Daily Menus. Per country it is shown how many foods complied with the Choices criteria, as well as the amount of foods that did not comply: The non-complying foods are divided in those that were either replaced, when an alternative was available on the market, or not replaced.

**Figure 3:**
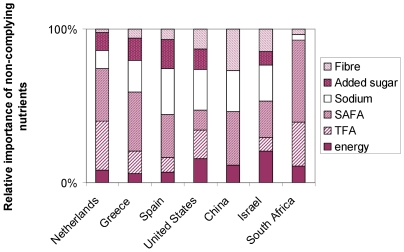
For the replaced foods: which key nutrient did not comply (% of total). Per country the relative importance of the non-complying key-nutrients in the replaced foods is shown. This is expressed as percentage of all non-complying nutrients. This is calculated for each key-nutrient by dividing “the amount of foods that were non-compliant for the key-nutrient” by “the sum of all non-complying key-nutrients for all foods”. It must be noted that foods can be non-compliant for more than one key-nutrient.

Figure 2 shows per country the absolute amount of compliant foods in the Typical Daily Menus and the amount of foods that was replaced. The Greek Typical Menus contained most compliant foods expressed as a percentage of the total amount of foods (59%) and the US Typical Menus the least (29%). In the Dutch Menus the largest percentage of foods was replaced (58%) and the Chinese Menus the smallest (20%). When considering the nutrient content, it is shown in Figure 3, that overall these replaced foods were mostly too high in SAFA and sodium although there are differences between countries. For example, TFA was the second most important reason to replace foods in the Dutch and the South African menus, while for the Chinese Typical Menus, there were no foods too high in TFA. The latter was the case for added sugar in China and South Africa.

## Results

Table 4 summarizes the overall results of the Daily Menu validation for the 7 different countries. Measured intakes from surveys, and calculated intakes (averaged for the three Typical Menus and the three Choices Menus) are shown together with the recommendations. Table 4 also shows the relative change in nutrient intake which occurs when non-compliant foods are replaced by foods that do comply with the Choices criteria.

**Table 4:** Overview results: Potential impact of Choices Programme on nutrient intakes as calculated by the Daily Menu Method (based on 3 daily menus) for 7 countries.

	Intakes	Energy (kcal/d)	SAFA (en%/d)	TFA (en%/d)	Sodium (mg/d)	Added Sugar (en%/d)	Fibre (g/d)
Recommendation		2000	10	1	2400	10	25
**Netherlands**	**Measured**	2190	14.2	1.7	2785*	15.5†	21.0
	**Typical**	2122	15.4	1.2	2753	13.0	18.3
	**Choices**	1788	8.4	0.1	2347	5.8	25.4
**% Change (Typical –Choices)**		−16	−45	−92	−15	−55	+39
**Greece**	**Measured**	2210	13.0	0.7	2125*		15.3
	**Typical**	2242	12.3	0.2	2029	10.6	16.4
	**Choices**	1867	8.7	0.1	1685	4.4	21.7
**% Change (Typical –Choices)**		−17	−29	−50	−17	−58	+32
**Spain**	**Measured**	2822	11.7	0.7	3600–4000‡		18.8
	**Typical**	2725	11.7	0.3	3608	7.2	20.5
	**Choices**	2252	6.9	0.2	2343	2.3	27.5
**% Change (Typical –Choices)**		−17	−41	−33	−35	−69	+34
**USA**	**Measured**	2146	11.2		3375	15.7	15.1
	**Typical**	2288	11.3	0.5	3522	13.9	17.6
	**Choices**	2110	6.6	0.2	2640	4.2	24.4
**% Change (Typical –Choices)**		−8	−42	−60	−25	−70	+39
**China**	**Measured**	2134	5.0§	0.2§	6008		11.1
	**Typical**	2106	8.7	0.1	5808¶ (1573)	0.1	12.6
	**Choices**	2055	5.6	0.1	5744¶ (1509)	0	20.3
**% Change (Typical –Choices)**		−2	−36	0	−1	−100	+61
**Israel**	**Measured**	1856	9.6		2816*		17
	**Typical**	1942	8.6	0.9	3072	8.6	17.2
	**Choices**	1653	5.4	0.3	2213	6.0	26.1
**% Change (Typical –Choices)**		−15	−37	−67	−28	−30	+52
**South Africa**	**Measured**	1990**	9.5††	0.7	3100‡	12.5‡‡	17.4
	**Typical**	2323	12.2	0.6	3026¶ (1600)	12.2	18.4
	**Choices**	2117	9.3	0.6	2804¶ (1378)	11.2	19.0
**% Change (Typical –Choices)**		−9	−24	0	−7	−8	+3

SAFA: saturated fatty acid; TFA: trans fatty acid.

*Measured sodium intakes for the Netherlands, Greece and Israel do not take into account discretionary salt.

† A translation for total sugars to added sugars has been applied by assuming that in general two-thirds of total sugars are delivered by added sugars.

‡ Data on total salt intake (including discretionary salt) from 24 h urinary sodium excretion [26], [34].

§ Data from the INTERMAP study (China) with 839 adults (aged 40–59) on nutrient intakes in the late 90es [25].

¶ Including discretionary sodium intake which is 4235 mg/day for the Chinese urban population & 1426 mg/d in South Africa. Between brackets: calculated sodium intake from the menus.

**Calculated average energy intake from multiple food consumption surveys [31].

†† Average of urban middle & urban upper class. Actual SAFA intake are estimated to be higher (12.2 en%) due to new insights on SAFA content of foods (unpublished results).

‡‡ Added sugar intakes were based on estimations form the South African Sugar Association (unpublished results).

The difference between the calculated nutrient intakes from the three Typical Menus and the measured intakes derived from the surveys, should ideally not be more than 20%. This could not be achieved for TFA (for The Netherlands, Greece, Spain, China) due to unavailability of food composition data. Measured intakes were not available for TFA (US, Israel) and added sugar (all countries except US). Therefore alignment of the nutrient intakes from the three Typical Menus with the reported measured intakes was not possible, except for the Netherlands where added sugar intakes were estimated. In general nutrient intakes as calculated from the Choices Menus moved in the direction of the recommendations. For all calculated intakes given in Table 4, excluding energy, the percentage of calculated nutrient intakes that were in line with the recommendations increased from 34% (for the Typical Daily Menus) to 77% (for the Choices Daily Menus).

### Energy

Table 4 illustrates that changes in energy intakes were moderate when regular foods that did not comply with the Choices criteria, were replaced by compliant foods. Change in energy intakes ranged between −2%, for China and −17% for Spain and Greece. Figure 3 confirms that energy is not in the top 3 of most critical nutrients in the replaced foods, with the exception of Israel. Typical foods that were too high in energy were beverages and snacks.

### SAFA

SAFA intakes from the Choices Menus were reduced towards recommendations, when compared with the Typical Menus (Table 4). Largest reductions are shown for The Netherlands (−45%), the US (−42%) and Spain (−41%). For China and Israel, SAFA intakes were already in line with recommendations, but they still decreased further (−36% and −37%, respectively). Also Figure 3 shows that SAFA was too high in many of the replaced foods. Examples of foods that were replaced are dairy, cheese, meats, fats, snacks.

### TFA

Intakes of TFA from the Choices Menus were also reduced towards recommendations as compared to the Typical Menus. This was the case for the Netherlands and also for Greece and Spain where typical TFA intakes were already below recommended levels. The large reductions in TFA intakes in the Netherlands (Table 4) are confirmed by Figure 3, where TFA was too high in a considerable percentage of the replaced foods. Examples of these foods were fats and snacks.

### Sodium

Table 4 illustrates that with the Choices Menus all sodium intakes reduced towards recommendations when compared to the Typical Menus. For Greece the typical sodium intakes were already in line with recommendations and were reduced further. For China and South Africa reductions were relatively small (−1% and −7%, respectively) and sodium intakes from the Choices Menus remained too high. The small reduction in Chinese sodium intakes was attributed to fish, and bread products and in South Africa to sauces. For the other countries, percentage reduction varied from −15% (The Netherlands) to −35% (Spain) and typical replaced foods that were too high in sodium were bread, cereals, sauces, meats and dairy.

### Added sugar

With the Choices Menus, overall added sugar intakes reduced towards recommendations (Table 4). For Spain, China and Israel typical added sugar intakes were already below recommendation. Figure 3 shows that none of the foods in the Chinese and the South African Typical Menus was replaced because of added sugar, either because added sugar levels were low (China) or no alternative was available on the market (South Africa). For the other countries typical high added sugar foods that were replaced were sweet snacks, cereals and also sugar in coffee (in Greece, where sweeteners are commonly used).

### Fibre

Intakes of fibre were increased (range from +3% in South Africa to +61% in China) for all countries when Choices Menus are compared to Typical Menus. However fibre intakes remain below recommendations in the Choices Menus for Greece, China and South Africa (Table 4). Typical replaced foods that were too low in fibre were bread, grain products and fruit juice.

## Discussion

The present article describes the Daily Menu Method as a tool to estimate the potential effects of nutrient profiles such as the Choices criteria, on daily nutrient intakes. Results demonstrate that calculated nutrient intakes move towards nutrient recommendations when regular non-complying foods were replaced with Choices-compliant foods in a Typical Daily Menu. This confirms that the criteria that have been set for Choices were strict enough to potentially move intakes into a direction that is more favourable for health. Calculated intakes of energy, SAFA, TFA, added sugar and sodium were reduced and fibre intakes were increased. This was the case for all countries for which this calculation was carried out, however the size of the effects differed between countries.

For all countries relative changes in energy intake were comparable and moderate. For SAFA intakes changes were also similar among the different countries, but reduced more profoundly even when SAFA consumption was already below the recommended limit (China and Israel). Changes in TFA and added sugar intakes varied between countries, possibly due to underestimation because of limited data availability or because (in China) TFA and added sugar consumption was already below recommended levels. Salt intakes were reduced but the magnitude of the effects strongly depended on the type of data. Highest relative reductions in salt intakes were shown when discretionary salt was included in the measured intakes and replaced. This was the case for Spain and the US. When discretionary salt intake was not replaced, the relative changes were small (China, South Africa). Overall fibre intakes were increased considerably. The small changes in fibre and also added sugar intake in South Africa were related to limited availability of alternative foods on the market (e.g. for white rice, flour, sugar).

The quality of the dietary surveys determined the quality of the intake data. In general nutrient intake data must be judged critically, because of underreporting and different sources of bias. Bias is related to e.g. the dietary assessment method used, selection of the population and availability of food composition data. Especially for TFA and added sugar, food composition data were not always available and hence were estimated.

Although it was the intention to perform the Daily Menu Method in a standardized way across the countries, this was not achieved entirely: For example, in the composition and calculation of the Chinese and South African Typical Menus, discretionary salt was not included in the menus. Therefore discretionary salt intakes could not change. Sodium intakes were measured differently as mentioned above, either as sodium excretion in 24 h urine, or via a dietary assessment method either with or without questions to determine discretionary sodium consumption.

Despite these limitations, it can be concluded that the Daily Menu Method allows a quantitative look into the calculated changes that nutrient profiles, such as those of the Choices Programme, can have on a countries' nutrient intakes. However, the method needs further standardisation, especially with respect to sodium intakes.

Other nutrient profiles that categorize foods based on nutrient content serve various goals: regulation of advertising [3], nutrition labels [47], [48], regulation of health claims [49] and product innovation tool [5], [50] and a number of them were developed in an international context [5], [9], [50]. Most systems are a translation from recommendations for total diet into recommendations for foods. Drewnowski and Fulgoni wrote in their review on nutrient profiling [12] that “ranking foods by their nutrient content is supposed to be a science and not an exercise in consensus building”. Thus nutrient profile models need to be evaluated and validated against an objective independent measure of diet quality, ideally in an international context [11].

One of the first approaches to evaluating nutrient profiles was presented by Azais-Braesco et al [51] using a classification of 125 food products. Foods were ranked by the various nutrient profiling systems and compared against the opinion of an expert nutritionist panel (n = 12). A similar exercise with a much larger group of 700 British nutrition professionals ranking 120 foods was carried out by Scarborough et al [52]. Although this method of “convergent” validation is simple and transparent, the authors noted that nutrition professionals are not entirely logical in their judgement, which will most likely also be influenced by cultural differences. A more internationally applicable validation method was developed by Volatier et al [53] in which “indicator foods” were derived from various national European surveys linked to healthier and less healthy dietary patterns. However, this method is subject to confounding and needs further refinement, as the authors stated themselves [53], [54]. Drewnowski & Fulgoni suggested to link nutrient profiles to measures of diet quality [12] as in the approach by Volatier et al [53] and more recently by Fulgoni et al [55]. Diet quality scores are developed via nutritional epidemiology to link dietary patterns rather than single dietary components, to health outcomes. However, in a critical review of predefined diet quality scores, it was concluded that these scores are not better than individual dietary factors [56]. Both diet quality scores and nutrient profiles are results of predefined decisions to include or exclude nutrients in the model to evaluate either diets or foods. Arambepola et al [57] described the use of such a diet quality measure in another approach towards validation of nutrient profiles: Again the “WXYfm” model developed for the UK FSA (United Kingdom Food Standards Agency) to regulate advertising to children, was validated against the food categorisation of the British food-based-dietary-guidelines [58] and also against healthiness of diets using the Diet Quality Index and the dietary intake data of the British National Nutrition Survey. The authors concluded that good “construct” validation was confirmed for the “WXYfm” model. However, there is limited international applicability of the validation method used. In addition, there is a certain circularity in reasoning, since healthiness of foods and diets both included measurements of nutrients such as SAFA and salt and ingredients such as fruits and vegetables [57]. This will be a general problem when using measures of diet quality for evaluation and validation of nutrient profiles. However, linear programming used to test the compatibility between nutrient profiling and nutrient-based recommendations as published by Darmon et al [10] might be a more objective approach.

Many of the validation methods are aimed at the question whether specific foods are categorized in the correct way as “healthier” or “less healthy”. Another way of approaching external validation is to estimate whether the nutrient profiles are suitable for their purpose or predict a future external outcome. One of the aims is to stimulate changes in nutrient intakes through nutrient profiles as reformulation targets. The Daily Menu Method estimates the potential effect on nutrient intakes of the nutrient profile system, in this case the criteria for the Choices logo. Results clearly show that calculated intakes shifted towards nutrient recommendations in the ideal situation when consumers choose healthier options that are available on the market. This indicates that the criteria for Choices are strict enough the have a potential effect. These results were confirmed in another study where statistical modelling was used to estimate potential effects on usual nutrient intakes if Dutch consumers would only choose foods that comply with Choices criteria [59]. It must be noted that these are potential effects and it remains to be seen whether consumers would really make these dietary changes.

An advantage of the Daily Menu evaluation method is the international applicability and a lack of circularity. The criteria of Choices are independent of the nutrient intake data on which the Daily Menus were based. However also in this method there is still a subjective judgement needed from a nutritionist/dietician to compose menus based on expert knowledge of dietary habits of the specific country and replacement of certain foods with others. In addition, standardization is important and could be improved.

In summary, from these Daily Menu evaluations of the potential impact on nutrient intakes of the Choices Programme, it can be concluded that the Daily Menu Method can be an effective approach to evaluate nutrient profiles. It would be valuable to have Daily Menu calculations for more countries across the world. In a next step it is recommended to further standardize the method, especially with respect to dealing with missing data, the different measures of sodium intakes and composing the menus and replacing the foods. It is also recommended to include a broader range of nutrients (including protein, vitamins and minerals) and compare the different nutrient profiling methods that exist worldwide.

## Supporting Information

Table S1 The Netherlands: Calculations of Daily Menus. Detailed calculations of three Typical Daily Menus and three Choices Daily Menus for The Netherlands.
(0.16 MB XLS)

Table S2 Greece: Calculations of Daily Menus. Detailed calculations of three Typical Daily Menus and three Choices Daily Menus for Greece.
(0.15 MB XLS)

Table S3 Spain: Calculations of Daily Menus. Detailed calculations of three Typical Daily Menus and three Choices Daily Menus for Spain.
(0.16 MB DOC)

Table S4 USA: Calculations of Daily Menus. Detailed calculations of three Typical Daily Menus and three Choices Daily Menus for the USA.
(0.13 MB XLS)

Table S5 China Caluclations of Daily Menus. Detailed calculations of three Typical Daily Menus and three Choices Daily Menus for China.
(0.16 MB XLS)

Table S6 Israel: Calculations of Daily Menus. Detailed calculations of three Typical Daily Menus and three Choices Daily Menus for Israel.
(0.14 MB XLS)

Table S7 South Africa: Calculations of Daily Menus. Detailed calculations of three Typical Daily Menus and three Choices Daily Menus for South Africa.
(0.14 MB XLS)

Table S8 Sources of food composition data and foods that could not be replaced. Overview of the food composition data sources that were used. In addition the foods that could not be replaced are given.
(0.05 MB DOC)
