# Supplementary material for: Potential Effects of Nutrient Profiles on Nutrient Intakes in the Netherlands, Greece, Spain, USA, Israel, China and South-Africa
Source: PLoS One. 2011 Feb 23;6(2):e14721. doi: 10.1371/journal.pone.0014721 (PMC3044133; doi:10.1371/journal.pone.0014721)
Supplement: Table S8 — Sources of food composition data and foods that could not be replaced. Overview of the food composition data sources that were used. In addition the foods that could not be replaced are given. (0.05 MB DOC) [file pone.0014721.s008.doc]

**Table S8. Sources of food composition data and foods that could not be replaced**

| **Country** | **Main data source** | **Additional data sources** | **Foods that could not be replaced, due to unavailability of a suitable alternative** |
| --- | --- | --- | --- |
| **Netherlands** | Dutch food composition database (1) | Data on added sugar content is estimated, based on the USDA database for added sugars (2) and some data for snacks from producer’s internet sites (3) | Boiled egg; potato chips; cracker (knackerbrod); breakfast cereals (muesli) |
| **Greece** | Greek food composition database (4) | Data on added sugar content is estimated, based on the USDA database for added sugars (2)  TFA values are estimated based on the McCance & Widdowson food composition table (5).  Additional information was found on commercial websites accessed through the Global New Product Database (3) | Honey; sesame bagel; corn flakes; lentils soup; spinach-cheese pie; olives; trahana soup; vanilla cake; egg omelette |
| **Spain** | Spanish food composition database (6). | Added sugars were estimated either as 0% or 100% of total sugars: cocoa, biscuits, cola drink, mayonnaise, sugar, cereals, milk chocolate.  TFA values are estimated based on the McCance & Widdowson food composition table (5).  Additional information was found on commercial websites accessed through the Global New Product Database (3) | Walnuts; eggs; milk chocolate, olives; white bread; jam; biscuits; chocolate chip cookies |
| **USA** | US food composition database (7) | Data on added sugar content was used from the USDA database for added sugars (2)  TFA values were taken from the US table for “Fat and Fatty Acid Content of Selected Foods” (8) Additional information was found on commercial websites accessed through the Global New Product Database (3) | Orange juice drink; potato chips, plain, salted; salad dressing 1000 island; ham sliced regular (approximately 11% fat); cheddar cheese; scrambled eggs; tomatoes cooked stewed |
| **China** | Chinese food composition database 2002 (9) | Data on added sugar content is estimated, based on the USDA database for added sugars (2)  TFA values are estimated based on the McCance & Widdowson food composition table (5).  Additional information was found on commercial websites accessed through the Global New Product Database (3) | Egg; soy sauce; vinegar; rice (white); wheat bun leavened steamed processed; pork tenderloin (raw). |
| **Israel** | Israel food and nutrient database (10) | Data on added sugar content is estimated, based on the USDA database for added sugars (2)  Additional information was found on commercial websites accessed through the Global New Product Database (3) | White rice; egg; cooked vegetables (salt); hummus; pita bread; biscuits; couscous; falafel |
| **South Africa** | South African food composition database (11) | Data on added sugar content is estimated, based on the USDA database for added sugars (2)  TFA values are estimated based on the McCance & Widdowson food composition table (5).  Additional information was found on commercial websites accessed through the Global New Product Database (3) | Sugar; maize meal porridge; egg; beef chuck; white rice; tomato onion curry sauce; flour; non-dairy coffee creamer; beef extract (spread); mutton chops |

TFA: trans fatty acid

1. Stichting Nederlands Voedingsstoffenbestand (2006) NEVO food composition database. Den Haag: Voedingscentrum.
2. U.S. Department of Agriculture (USDA) (2005) USDA database for the added sugars content of selected foods. Available: <http://www.ars.usda.gov/SP2UserFiles/Place/12354500/Data/Add_Sug/addsug01.pdf Accessed 2010 Nov 30>.
3. Global New Product Database MINTEL. Available: www.gnpd.com. Accessed 2009, May 15.
4. Trichopoulou A (2007) Composition tables of foods and Greek dishes' (3rd edition). Available: [www.nutrition.med.uoc.gr/GreekTables/Main/main.htm. Accessed 2008 Oct 15](http://www.nutrition.med.uoc.gr/GreekTables/Main/main.htm. Accessed 2008 Oct 15).
5. Foods Standards Agency. (2002) McCance and Widdowson's the Composition of Foods: Summary Edition.
6. Farran A, Zamora R, Cervera P (2004) Food Composition Tables of CESNID (The Centre of Superior Studies in Human Nutrition and Dietetics). Ed.:McGraw-Hill/Interamericana de España, S.A.U. Barcelona: University Editions.
7. U.S. Department of Agriculture, Agricultural Research Service (2008) USDA National Nutrient Database for Standard Reference, Release 21. Nutrient Data Laboratory. Available: <http://www.ars.usda.gov/ba/bhnrc/ndl>. Accessed 2009 Oct 15.
8. US Department of Agricultural Research Service (2008) USDA Database. Fat and Fatty Acid Content of Selected Foods Containing Trans-Fatty Acids 1989-1993. Available: http://www.ars.usda.gov. Accessed 2008 Jan 15.
9. Yang Y, Wang G, Pan X, editors (2002) China Food Composition Table 2002. The Institute of Nutrition and Food Safety, Chinese Center for Disease Control and Prevention. China: Peking University Medical Press.
10. Food and nutrition services and public health services Israel Ministry of Health (2008) Israeli Nutrient Database (BINAT). Derived from Tzameret software on consumption of food and nutrients.
11. Medical Research Council. (2002) Foodfinder 3. Food composition analysis software. Available: http://foodfinder.mrc.ac.za/ Accessed 2010 Nov 30.
